# Provision of somatosensory inputs during motor imagery enhances learning-induced plasticity in human motor cortex

**DOI:** 10.1038/s41598-017-09597-0

**Published:** 2017-08-24

**Authors:** Gaia Bonassi, Monica Biggio, Ambra Bisio, Piero Ruggeri, Marco Bove, Laura Avanzino

**Affiliations:** Department of Experimental Medicine, section of Human Physiology, University of Genoa, Genoa, Italy

## Abstract

Motor learning via physical practice leads to long-term potentiation (LTP)-like plasticity in motor cortex (M1) and temporary occlusion of additional LTP-like plasticity. Motor learning can be achieved through simulation of movement, namely motor imagery (MI). When combined with electrical stimulation, MI influenced M1 excitability to a larger extent than MI itself. We explored whether a training based on the combination of MI and peripheral nerve stimulation (ESMI) modulates M1 LTP-like plasticity inducing retention of a new acquired skill. Twelve subjects mentally performed thumb-index movements, with synchronous electrical nerve stimulation, following an acoustic cue, in order to increase movement speed. Two control groups physically performed or imagined the same number of finger movements following the acoustic cue. After each training session, M1 LTP-like plasticity was assessed by using PAS25 (paired associative stimulation) technique. Performance was tested before and after training and 24 hours after training. Results showed that physical practice and ESMI training similarly increased movement speed, prevented the subsequent PAS25-induced LTP-like plasticity, and induced retention of motor skill the following day. Training with MI had significant, but minor effects. These findings suggest that a training combining MI with somatosensory input influences motor performance through M1 plasticity similarly to motor execution.

## Introduction

Motor practice leads to acquisition or improvement of kinetic skills. With physical exercise, movements become faster, more accurate and effortless (i.e., motor learning)^1^. Motor learning through repetition of a movement leads to a long-term potentiation (LTP) of the primary motor cortex (M1) in humans as in animals^2^. This phenomenon of LTP leads to a temporary occlusion of M1 to further potentiation, according to the concept of homeostatic plasticity^3–6^. Evidence in the literature suggests that in humans, the occlusion of LTP-like plasticity after learning, indicative of how much LTP was used to learn, is essential for retention^7, 8^.

Paired associative stimulation (PAS) is a transcranial magnetic stimulation (TMS) protocol able to induce LTP-like effects in M1^9, 10^. PAS25 consists of pairs of electrical stimulation of the median nerve, followed by TMS of the hand area of the contralateral M1 at an interstimulus interval of 25 ms. PAS25 leads to an LTP-like long-lasting increase in MEP amplitude of a median nerve innervated hand muscle^11^.

Noteworthy, other TMS techniques are able to induce LTP-like plasticity in M1, such as repetitive TMS or theta-burst stimulation. These techniques imply the application of train of TMS stimuli over M1 in a regular fashion (trains of TMS stimuli repeated at frequencies higher than 1 Hz) or in a patterned fashion (50-Hz triplets repeated at 5 Hz). PAS on one side, and theta burst stimulation and repetitive TMS on the other, differ particularly in the role of sensory input, which is relevant in PAS, but absent in theta burst stimulation and repetitive TMS^12^. Indeed, PAS25 is a plasticity induction protocol that is dependent on sensory afferent stimulation. For this reason, PAS25-induced plasticity shares similarities with experimental protocols inducing synaptic “spike timing-dependent plasticity” *in vitro* and *in vivo* ^12, 13^. Thus, PAS25 has been showed to be the most appropriate protocol to use when testing M1 plasticity induced by sensory afferent stimuli or by inputs to M1 coming from neural structures influencing sensory processing, including cerebellum^14^.

Motor learning is also achievable without moving, with internal simulation of the movement, namely motor imagery (MI). Actual and imagined movements trigger similar motor representation and share similar brain substrates^15, 16^. Particularly, imagery-related activity is in general more closely related to instruction-related activity (motor planning phase) than to motor execution-related activity^17^. In a recent study we investigated M1 plasticity induced by physical or motor imagery practice^18^. Both protocols were able to improve motor performance, even if improvement was greater after physical practice than after motor imagery practice. Furthermore, an increase in M1 cortical excitability was present after physical practice, but not after motor imagery practice. Finally, both protocols led to the development of neuroplasticity, as they affected the PAS25- induced plasticity in M1, but observed effects after physical practice were stronger than after motor imagery practice. We explained these findings as related to different sensorimotor mechanisms operating during the two training methods. Indeed, one of the main differences between physical and motor imagery practice is the lack of somatosensory afferent inputs in the imagined movements.

It has been recently demonstrated that the association of motor imagery and peripheral nerve electrical stimulation could enhance cortico-spinal excitability during MI practice, to a larger extent with respect to peripheral nerve electrical stimulation or MI alone^19, 20^. Particularly, the combination of the activation of the internal model of motor commands, due to the MI, and the external activation of afferent input, given by peripheral nerve electrical stimulation led to a similar increase of the cortico-spinal excitability as real movement.

Here we explored whether a training session based on the combination of motor imagery and peripheral nerve electrical stimulation leads to (i) occlusion of LTP-like plasticity in M1 and (ii) retention of skill learning likewise physical practice. To test for LTP-like plasticity we adopted the PAS 25 protocol, since, as stated above, it is the most suitable non-invasive brain stimulation protocol to test for plasticity induced by sensory stimulation.

To this aim, we assessed LTP-like plasticity in M1 and retention of motor skill induced by motor learning through (i) motor imagery combined with peripheral nerve electrical stimulation; (ii) motor imagery alone and (iii) physical practice training sessions. We hypothesized that learning through motor imagery combined with peripheral nerve electrical stimulation would be more efficient than MI alone in inducing occlusion of LTP-like plasticity and retention of motor skills, which is known to be dependent on the occlusion of LTP-like plasticity.

## Results

### Experimental design

Forty-four right-handed subjects (mean age 24.97 ± 4.99, 18 males) participated in this study. All participants took part to a first experimental session (Day 0) designed to test the effect of PAS25 on motor evoked potentials (MEPs). Subjects were admitted to the subsequent experimental sessions if, in the first session, PAS25 had led to a significant increase of MEPs amplitudes. To control for it, we recorded twenty MEPs from the target muscle before and after a 30 minutes -PAS25 protocol on the homologous motor cortex. MEPs data collected before and after PAS 25 were compared by means of a paired Student t test. Only participants in which statistical analysis between MEPs before and after PAS25 yielded a statistically significant increase at a *p* < 0.05 were admitted to the subsequent sessions.

**Figure 1:**
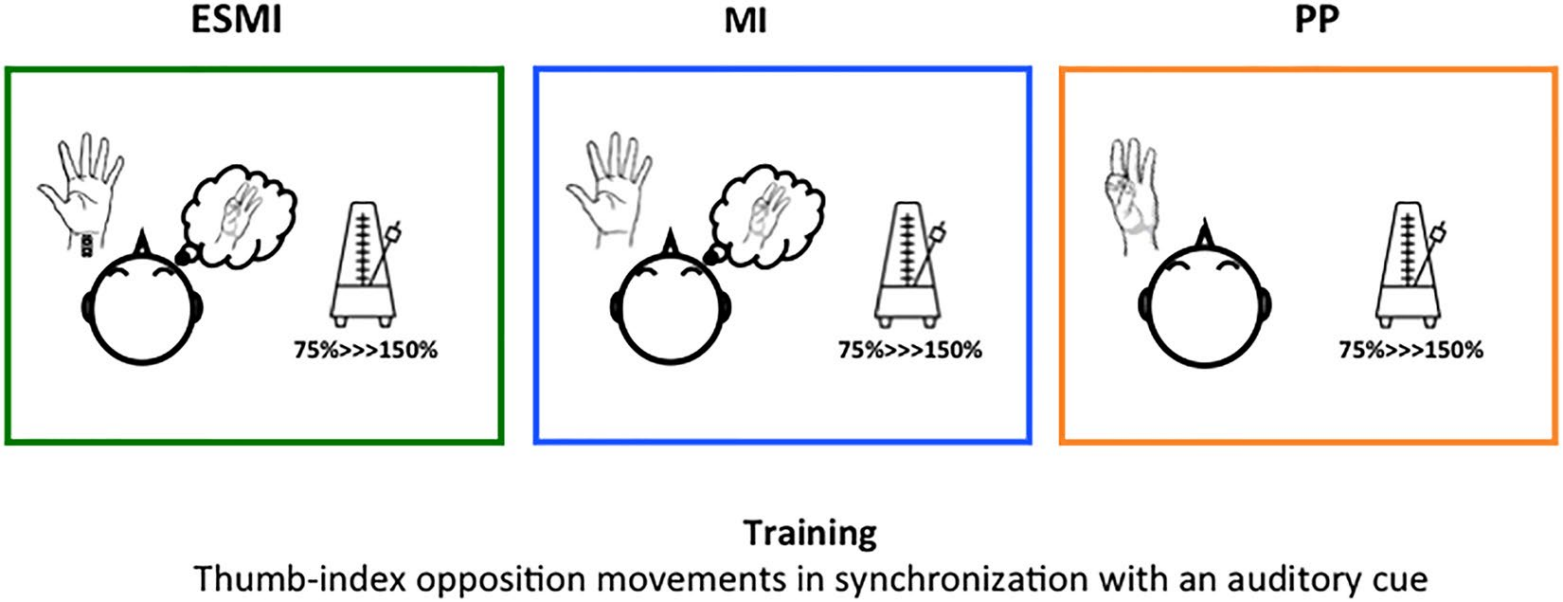
Training sessions. During the Motor Imagery and Electrical Stimulation (ESMI) training session, subjects had to imagine a thumb-index opposition movement following a metronome while receiving a synchronized electrical stimulus on their median nerve. During physical practice (PP) or motor imagery (MI) training session, subjects had to execute or to imagine (kinaesthetic motor imagery) the thumb-index opposition movement following the acoustic cue. The acoustic cue was set at increasing frequencies (from 75% to 150% of individual maximal finger movements rate).

Of 44 subjects, 36 fulfilled this criterion and were randomly divided in three groups for participating to the next experimental sessions (Day 1–Day 2), separated by at least 1 week from the first one. The three groups were matched for age and gender distribution. On Day 1, participants trained a task of thumb-index opposition with their non-dominant hand, in order to increase their movement rate. The experimental groups differed in terms of the type of training performed. The main experimental group (12 subjects) executed the Motor Imagery and Electrical Stimulation (hereafter defined by ESMI) training session, during which they had to imagine a thumb-index opposition movement (kinaesthetic motor imagery) while receiving a synchronized electrical stimulus on their median nerve. Two different groups formed by 12 subjects each, executed a physical practice (hereafter PP) or a motor imagery (MI) training session, during which they had to execute or to imagine (kinaesthetic motor imagery) the thumb-index opposition movement. Noteworthy, during training all subjects were instructed to imagine or perform thumb-index opposition movements following a rhythmic acoustic cue. Acoustic cue was set at increasing frequencies ranging from 75% to 150% of individual maximal finger movements rate. Precisely, given the high rate of the acoustic cue, and the subsequent difficulty to imagine the complete movement, subjects were instructed to match the instant of contact between thumb and index to the acoustic cue. This procedure allowed us to synchronize the electrical stimulus to the imagined movements in ESMI training since electrical stimulus was delivered simultaneously to the acoustic cue (corresponding to the thumb-index contact phase of the imagined movement) (Fig. 1).

**Figure 2:**
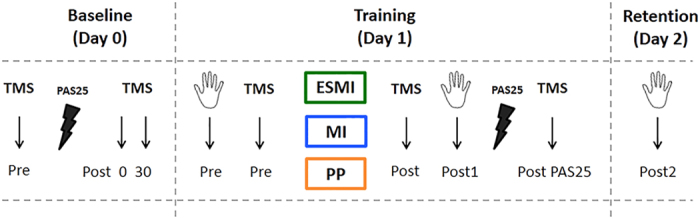
Experimental protocol. On Day 0 we tested the effect of Paired Associative Stimulation (PAS25) protocol on motor evoked potentials (MEPs) by means of transcranial magnetic stimulation (TMS). On Day 1 participants were divided into three groups, performing different trainings. We tested the effect of different trainings on cortical excitability and on PAS25 induced-effects. Moreover we tested the effect of training on finger movement’s performance through an engineering glove, immediately after the training session and the following day (Day 2). MEPs, motor evoked potentials. ESMI, Electrical Stimulation and Motor Imagery; MI, Motor Imagery; PP, Physical Practice.

Cortical excitability and thumb-index opposition performance at maximal speed was assessed before (Pre) and after (Post) the training in each group. Further, immediately after the training sessions, the PAS25 protocol and the subsequent measurement of MEPs were applied to test for LTP-like plasticity occlusion in M1. The following day in the retention session (Day 2), participants returned for performing thumb-index opposition task at maximal speed (Post2) (see Fig. 2 for the experimental protocol).

### Preliminary findings

**Figure 3:**
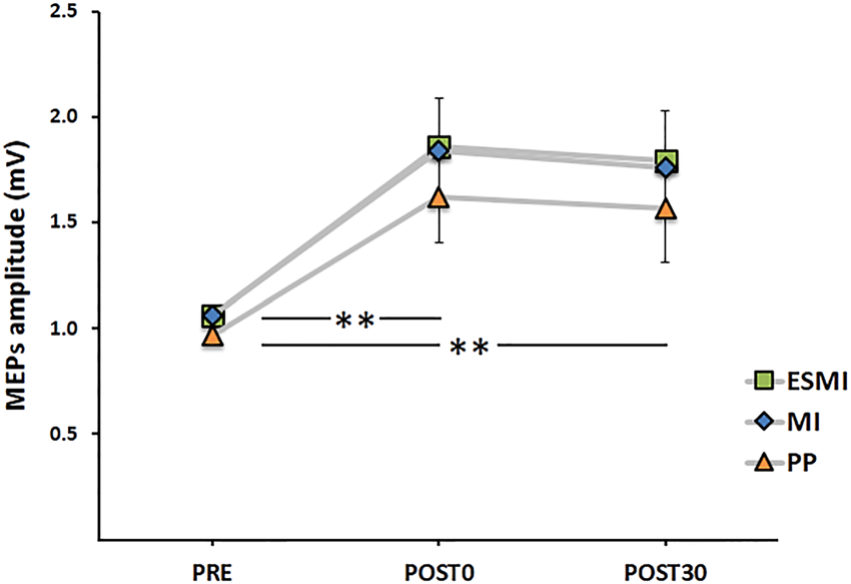
PAS25 results on Day 0. Amplitude of the motor-evoked potentials (MEPs) recorded before (PRE), immediately after (POST0), and 30 minutes (POST30) after the PAS25 protocol in the three groups. ESMI, Electrical Stimulation and Motor Imagery; MI, Motor Imagery; PP, Physical Practice.

Data from the Day 0 evaluation (Fig. 3), assessing the effect of the PAS25 protocol on subjects’ plasticity, showed a significant effect of time (F_2,66_ = 40.15; p < 0.001). Post hoc analysis revealed an increase of MEPs amplitude after the PAS25 protocol (PRE vs POST0, p < 0.001), maintained until 30 minutes after PAS25 application (PRE vs POST30, p < 0.001). No significant effect of GROUP or GROUP*TIME interaction were found.

### Motor performance

Participants were trained to physically or mentally (with or without peripheral nerve electrical stimulation) perform a task of thumb-index opposition, in order to increase their movement rate. We considered the mean movement rate value between the two performances executed in the assessment phase before the training (Pre), immediately after the training (Post1) and the following day (Post2).

Further we also normalized movement rate data collected after training and 24 hours later respect to baseline assessment. Finally, to measure individual accuracy in increasing movement rate after training with respect to the acoustic cue provided during training, we calculated, the difference between the time interval between two successive acoustic cues provided by the metronome set at 150% of maximal voluntary rate and the time interval between two successive movements reproduced by the subjects immediately after the training and 24 hours later (temporal accuracy index).

**Figure 4:**
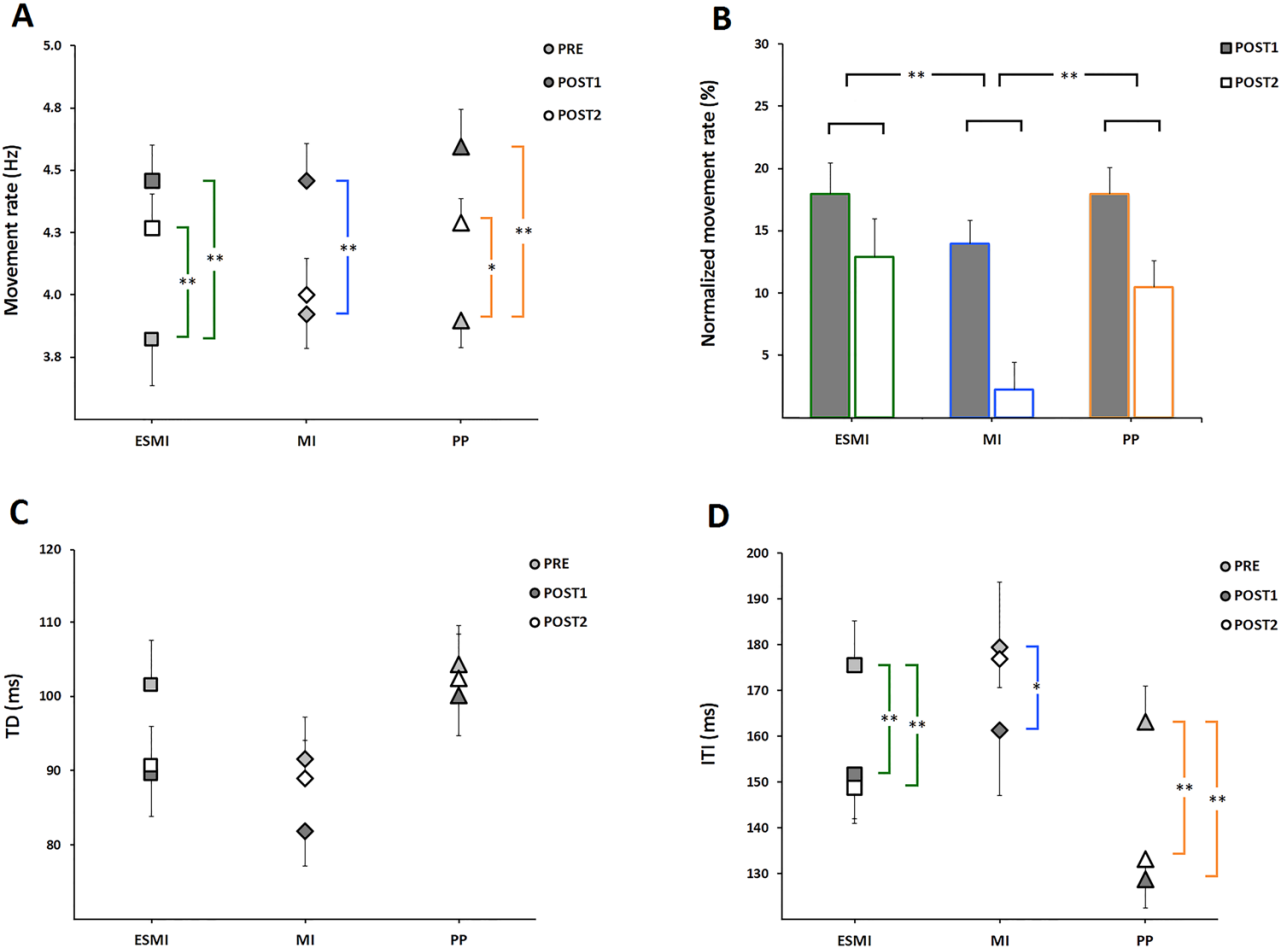
Motor performance. The effect of the different trainings on movement rate (**A**,**B**), touch duration (TD, panel C) and inter-tapping interval (ITI, panel D) is reported. In panel A-C-D, squares represent subjects of ESMI, diamonds represent MI subject and triangles correspond to PP subjects. In panel B the % change in movement rate is reported on Post1 (calculated as (Post1 − Pre)/Pre × 100) and on Post2 testing times (calculated as (Post2 − Pre)/Pre × 100). Vertical bars indicate standard error of the mean (SEM). Asterisks indicate the level of significance (*p < 0.05; **p < 0.01).

Statistical analysis on raw movement rate data showed a significant time *group interaction (F_4,66_ = 2.98; p = 0.025). Post hoc analysis revealed that movement rate increased after training in all the experimental group (Pre vs Post1, p always < 0.001), but whereas this increase was maintained 24 hours later in ESMI (Pre vs Post2, p < 0.001) and PP groups (Pre vs Post2, p = 0.001), in the MI group movement rate returned comparable to baseline values (Pre vs Post2, p = 0.36) (Fig. 4A).

Further, when we compared normalized movement rate data, a significant effect of group (F_2,33_ = 3.10; p = 0.045) was found. Post hoc analysis showed that movement rate changes in Day 1 and Day 2 were similar in the ESMI and in physical practice conditions (ESMI vs PP, p = 0.70) whereas the increase in movement rate after training on Day 1 and the retention of the acquired skill on Day 2 were smaller in the MI group respect to the other two (MI vs ESMI, p = 0.029; MI vs PP, p = 0.040) groups (Fig. 4B). This finding was also confirmed by statistical analysis on individual accuracy respect to the acoustic cue provided during the training. Indeed, RM-ANOVA showed a significant effect of group (F(_2,33_) = 4.83, p = 0.014). ESMI and PP group were more accurate, showing a smaller temporal accuracy index on Day 1 (ESMI, 41.78 ± 9.73 ms; PP, 45.94 ± 11.06 ms) and on Day 2 (ESMI, 54.01 ± 12.74 ms; PP, 58.14 ± 12.61 ms) respect to MI (Day 1, 50.02 ± 11.57 ms; Day 2, 68.37 ± 8.08 ms) (ESMI vs MI, p = 0.004; PP vs MI, p = 0.04).

When we analysed the kinematic parameters of finger movements, statistical analysis showed that the touch duration (TD, i.e., the time spent in the contact between the thumb and the index) decreased with training in all groups (time, F_2,66_ = 9.09; p < 0.001) immediately after training (Pre vs Post1, p < 0.001) and returned to baseline values 24 hours later (Pre vs Post2, p = 0.053) (Fig. 4C).

Differently, related to inter-tapping interval (ITI), i.e. the time elapsing from end of contact between the thumb and the index and the beginning of the subsequent contact, statistical analysis showed a significant group*time interaction (F_4,66_ = 2.90; p = 0.028). Post hoc analysis revealed that in ESMI and PP groups ITI decreased immediately after training (ESMI, Pre vs Post1, p = 0.002; PP, Pre vs Post1, p < 0.001) and 24 hours later (ESMI, Pre vs Post2, p < 0.001; PP, Pre vs Post2, p < 0.001) (Fig. 4D). Differently, in MI group ITI decreased immediately after training (Pre vs Post1, p = 0.018), but returned to baseline values 24 hours later (Pre vs Post2, p = 0.72) (Fig. 4D).

### Effect of different trainings on PAS-induced effects

**Figure 5:**
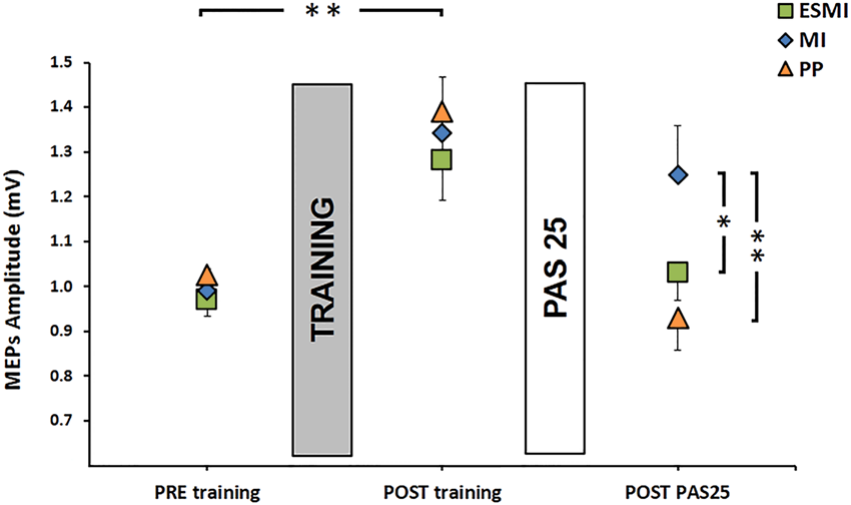
Effect of training on PAS25 in the three groups. Cortical excitability data of the three groups (ESMI, MI, PP) are shown. Squares represent subjects of ESMI, diamonds represent MI subject and triangles correspond to PP subjects. MEPs amplitude, in mV, is depicted before and after each training session (dark grey bars) and after PAS25 plasticity protocol (light grey bars). Vertical bars indicate standard error of the mean (SEM). Asterisks indicate the level of significance (*p < 0.05; **p < 0.01).

Regarding cortical excitability data, RM-ANOVA showed a significant effect of time (F_2,66_ = 35.83, p < 0.001), with a significant group x time interaction (F_4,66_ = 3.98, p = 0.006). Post hoc analysis revealed that MEPs amplitude increased immediately after training after ESMI training (ESMI: PRE training vs POST training, p = 0.001) as well as after physical practice (PP: PRE training vs POST training, p < 0.001), and motor imagery training (MI: PRE training vs POST training, p < 0.001) (Fig. 5). No differences between groups were found regarding MEPs amplitude on the POST training testing time (ESMI POST training vs MI POST training, p = 0.69; ESMI POST training vs PP POST training, p = 0.48; MI POST training, vs PP POST training, p = 0.75). Further, we found a significant correlation between MEPs amplitude increase after PAS25 on Day 0 and MEPs amplitude increase on Day 1, after training session in all groups (ESMI, r = 0.64, p = 0.026; PP, r = 0.65 p = 0.021 MI, r = 0.72 p = 0.007), indicating that in all groups “better” PAS-responders showed a stronger increase in MEPs amplitude immediately after training.

After the PAS25 protocol, groups that performed ESMI and PP training showed a significant decrease in MEPs amplitude (ESMI: POST training vs POST PAS25 p = 0.001; PP: POST training vs POST PAS25 p < 0.001), without differences between the two training protocols (POST PAS25: ESMI vs PP p = 0.41), whereas when PAS25 was applied after motor imagery training MEPs amplitude did not change (MI: POST training vs POST PAS25 p = 0.17) (Fig. 5). After the administration of the PAS25 protocol (POST PAS25 testing time) MEPs amplitude in the MI group was significantly larger than MEPs amplitude in the ESMI group (POST PAS25: MI vs ESMI, p = 0.045) and in the PP group (POST PAS25: MI vs PP, p = 0.013).

## Discussion

The main findings of this study include the following: (1) training through physical practice (PP) and motor imagery combined with peripheral nerve stimulation (ESMI) similarly induced motor learning, more than training through motor imagery alone (MI); (2) the three types of training (ESMI, MI or PP) induced an increase in cortical excitability; (3) all the training sessions (ESMI, MI or PP) prevented the subsequent PAS25-induced LTP-like plasticity, but the occlusion of LTP-like plasticity was stronger after both the ESMI and physical practice training sessions than after motor imagery alone; (4) this neurophysiological finding was paralleled by better retention of the newly acquired skill, reflected by performance in the second day of practice, in the ESMI and PP groups with respect to MI group.

Our behavioural data showed that after training, motor performance improved in all groups, even if improvements observed after ESMI and PP trainings were stronger than after MI training, in accordance with previous studies^18, 21, 22^. Further, retention of the acquired skill, tested as motor performance on the subsequent day, was present in ESMI and PP groups but not in MI group. Particularly, the decrease in the ‘inter tapping interval’, that can be interpreted as a pure ‘motor time’ during finger opposition movements, was maintained until 24 hours after training in the ESMI and PP groups.

As already discussed in a previous study^18^, differences in motor learning between motor imagery and physical practice may be explained by different sensorimotor mechanism used during training. While physical practice involves both motor and sensory processes in order to improve the quality and the efficiency of the movement, training through imagination rests on internal forward models, which predict the future state without any bottom-up feedback. Because of this difference, practice is probably less accurate in MI, leading to a smaller improvement in motor performance^18, 21, 22^. Recent evidence in the literature showed that the combination of MI and peripheral nerve electrical stimulation above motor threshold was able to influence M1 excitability similarly to voluntary movement^19, 20^. However no behavioural data are available in the literature so far on the efficacy of combined MI and peripheral stimulation training. Our behavioural findings show that when participants received a sensory feedback combined with the movement imagination during training, they improved their behavioural performance to the same extent as participants who executed the physical practice training.

Somatosensory feedback is able to redefine many aspects of a motor pattern, such as movement accuracy, pattern frequency and force adjustments for on-going movements^23–25^. Accordingly, a reduction of somatosensory inputs by short-term immobilization of a limb impairs motor performance, even if this modification quickly decreases during trial-by-trial movement repetition^26^. Short-term limb immobilization also affects cortical excitability and plasticity of the motor cortex contralateral to the restricted limb^27–31^, and these modifications are strongly dependant from the deprivation of sensory inputs. Indeed, these cortical effects were partially counteracted when somatosensory inputs were delivered to the restricted limb during the immobilization period^28^, supporting the general idea that sensory inputs crucially shape somatosensory networks. Accordingly, the provision of motor imagery by itself was not able to cope with the corticomotor depression induced by immobilization^32^.

Here, regarding M1 excitability, we observed an increase in MEPs amplitude after all the different training sessions. We decided a priori not to include a training with peripheral stimulus alone since it has already been showed that it doesn’t lead to any modification of M1 neuroplasticity^33^, unless when provided at higher frequencies or for longer stimulation periods^34–36^. The increase of M1 excitability induced by the “augmented” MI training was similar to that observed after physical practice. In contrast with our previous study^18^ and others^35^ the increase of M1 excitability after motor imagery training was similar to that observed after physical practice. However we think that a possible explanation of this finding could deal with the difference between the tasks adopted for the motor imagery training in these studies. Indeed, at difference with our previous study, subjects were asked to imagine thumb-index opposition movements following a rhythmic external auditory cue. Auditory cues have already been used to improve motor control and motor learning and the association of an external auditory cue with physical practice has been proven to promote motor skill acquisition and to get sport performance more efficient^37–39^. Moreover, the use of auditory cues significantly influences motor imagery increasing subjects’ imagery vividness, likely by triggering a separate neural system, the cerebello-thalamo-cortical, preferentially used in movement based upon external sensory cues^40^. Here we also showed that “better” PAS25 responders in the three groups were those subjects who showed a larger increase in M1 excitability immediately after the training session. These data confirm data in the literature suggesting that PAS25 and motor training by physical practice are likely to induce LTP-like plasticity on the same neural population in M1^41–44^, and enlarge this concept to different types of training like motor imagery training and training combining motor imagery and electrical stimulation.

Concerning the PAS25 effect on M1 excitability after training, in accordance with strong evidence in the literature, we found that physical practice prevented the subsequent PAS25-induced LTP-like plasticity in M1^41, 42^. Several studies showed that in humans, learning and non-invasive brain stimulation protocols, which individually are able to induce LTP-like plasticity in M1, interact with each other. Indeed, for the link between synaptic plasticity and memory formation to be confirmed, the principle of occlusion is required^45^; in other words, saturation of synaptic plasticity in a network should occlude new memory encoding^45^. This principle has been demonstrated for motor learning when interacting with non-invasive brain stimulation protocols able to evoke long-lasting changes of M1 excitability, as for instance paired associative stimulation^41, 42, 44, 46^. We have however to mention that, even if there is robust evidence in supporting the interaction between PAS25 induced-plasticity and motor learning, the mechanisms of action of paired associative stimulation protocols are not completely clear, possibly involving cortical plasticity in other cortical areas and interconnected networks apart M1^47^ and not always following temporal “spike timing-dependent plasticity” rules, likely depending also on the specific neuronal populations stimulated and on the activity state of the cortex^48, 49^.

However, here we showed for the first time that a training based on the combination of MI and a somatosensory afferent stimulation was able to induce the same effect as physical practice did, preventing the subsequent PAS25-induced LTP-like plasticity, in a stronger manner than MI alone. Our findings are in accordance and expand those by Mrachacz-Kersting and coworkers (2012). These authors showed that concomitance between motor imagery and the ascending volley due to the peripheral nerve stimulation could lead to a significant increase in cortical excitability^35^. Our neurophysiological finding was paralleled by better retention of the newly acquired skill, as reflected by performance in the second day of practice, in ESMI and PP groups respect to MI group. Particularly, when we analysed the kinematic properties of finger opposition movements, we found that changes in the time dedicated to movement (inter-tapping interval, ITI) persisted until 24 hours after training in ESMI and PP groups, while changes in the time dedicated to the contact between thumb and index (touch duration, TD) were similarly observed in the three groups only immediately after training. Although ITI is likely to represent a pure motor phase, TD may be regarded as the combination of a sensory phase and a motor preparation phase in which the subsequent movement is correctly planned prior to execution. The selective decrease of ITI maintained until 24 hours after the training session in ESMI and PP groups may suggest that these practice sessions favoured the formation of a new motor memory. For physical practice it has already been demonstrated that the magnitude of occlusion of LTP-like plasticity after training (that is an index of the amount of LTP-like plasticity used during motor learning), was associated with better performance and more resilience to retrograde interference from a second task on a subsequent day, suggesting a retention mechanism^7^. Here we showed that similar mechanisms operated when learning is acquired through motor imagery training and can be enhanced through the provision of somatosensory inputs during motor imagery.

Further, our results fit in a novel scenario supporting that the combination of somatosensory inputs (provided by peripheral nerve stimulation) with cognitive processes of movement (motor imagery or action observation) could lead to strong changes in cortical excitability and motor performance. Indeed, when action observation was delivered in conjunction with a peripheral nerve stimulation, it induced an increase of the M1 excitability which outlasted the stimulation period, induced learning of a newly trained skill and prevented the subsequent induction of LTP-like plasticity^33, 50, 51^. Taken together this piece of evidence supports the use of protocols combining cognitive representation of movement and somatosensory inputs in order to induce LTP-like plasticity in M1 and learning and consolidation of a new motor skill.

Some issues still remain open and deserve to be explored in future studies. First, we a priori chose to test LTP-like plasticity with PAS25 protocol that is dependent on sensory afferent stimulation.

However, to better elucidate different mechanisms underlying LTP-like plasticity in M1 induced by motor imagery training (with or without electrical stimulation) it will be interesting to use different LTP-like plasticity induction protocols as theta burst stimulation or repetitive TMS. Second, on Day 2, we tested only for retention of motor skill learning, but in future studies it will be of interesting to assess also for long lasting changes in M1 excitability induced by different types of training.

In conclusion, our findings emphasize the role played by somatosensory inputs during motor imagery training. It has been suggested that brain activation during motor imagery likely corresponds to activation of the neural representations of a “potential” movement that is retrieved volitionally from motoric memory^17^. Here we showed that during motor imagery sensory feedback might be crucial in inducing LTP-like plasticity in M1. In other words, when combined with sensory stimulation, the cognitive retrieval of motor plan is able to induce plasticity in M1 (changes in synaptic efficiency) that in turns corresponds to motor learning and consolidation of a new motor memory. These results suggest combining motor imagery and somatosensory stimulation to induce motor learning, as in a rehabilitative setting or in sport.

## Materials and Methods

### Subjects

All participants were in good health, without any nervous, muscular, orthopaedic or cognitive disorders. Right arm dominance was determined by means of the Edinburgh Handedness inventory^52^. Participants’ general motor imagery ability was evaluated by means of the Italian version of the Movement Imagery Questionnaire (MIQ-R)^53^. The MIQ-R is an 8-item self-report questionnaire, in which participants rated the vividness of their mental representations using two 7-point scales, associated to kinaesthetic and visual imagery: the score “7” means “really easy to feel/see”, whereas the score “1” corresponds to “really difficult to feel/see” (best score = 56, worst score = 8). All participants showed good motor imagery abilities (mean ± SD: 45.81 ± 4.9). The experimental protocol was approved by the ethics committee of the University of Genoa and was carried out in agreement with legal requirements and international norms (Declaration of Helsinki, 1964). All subjects gave informed consent for participation in the study.

### Behavioral assessment

Subjects were seated on a chair wearing a sensor-engineered glove (Glove Analyzer System (GAS), ETT S.p.a., Italy) on their left hand^54^. We choose an eye-close paradigm to avoid confounding effects due to different kind of training (ESMI, MI, PP). To assess baseline performance, subjects had to execute a thumb-index opposition task at their maximal speed, two times, 30 seconds each, with 1 minute rest. We considered subjects average rate obtained from the two repetitions as individual 100% and we used this value to set the behavioral training. After each training session (Day 1) and one day later (Day 2), subjects repeated the thumb-index task two times 30 seconds each, in order to detect any speed change (Fig. 2). We a priori choose to assess and train the non-dominant side because the behavioral task demanded a movement speed increase as outcome measure of training and if the baseline frequency had been too fast (as for the dominant side in a very common task as thumb-index opposition movement), the amount of improvement would have been restricted from the physiological speed limit.

### Behavioral training

From the individual 100% finger movements maximal rate we calculated three further percentages: 75, 125 and 150%. During training, subjects had to mentally perform (ESMI, MI) or execute (PP) the thumb-index opposition movements following the rhythm marked by a metronome, adjusted on the four percentages calculated before. The finger opposition task was executed (or imagined) for 20 seconds, two times for each percentage, from 75% to 150% of the individual maximal rate, for a total of 8 repetitions. Particularly, given the high rate of the acoustic cue subjects were instructed to match the instant of contact between thumb and index to the acoustic cue. Even though the aim of the training was that of raising the frequency of the thumb-index opposition movements, during the training sessions we initially set the metronome on 75% of subject’s maximum speed, in order to allow subjects to familiarize with the acoustic cue and with imagined movement in the ESMI and MI groups. In ESMI training, participants had to kinaesthetically imagine the finger opposition task in rhythm with the metronome that, in turn, was synchronized with an electrical stimulus delivered over the left median nerve. Electrical stimulus was delivered simultaneously to the acoustic cue (corresponding to the thumb-index contact phase of the imagined movement. Electrical stimulation was delivered through a bipolar electrode over the left median nerve at the wrist (cathode proximal, constant square wave current, duration 200 microseconds, intensity set just above threshold for evoking a small twitch in the opponens pollicis muscle) (Digitimer D180 high voltage electric stimulator). In MI training participants were asked to kinesthetically imagine the same movement, without the peripheral stimulus, whereas in the PP training they had to physically execute the task, always following the metronome during the training session (Fig. 1).

### Transcranial magnetic stimulation (TMS)

Single-pulses were delivered using a Magstim 200 stimulator (Magstim Co., Whitland, Wales, UK) with a monophasic current waveform connected to a figure-of-eight-shaped coil (external diameter of each loop, 9 cm) held tangentially to the scalp. The center of the junction of the coil was placed over the hand area of the right M1 at the optimal position (hot spot) to elicit MEPs in the non-dominant FDI, with the handle pointing backwards and ~45° away from the midline. The optimal coil location was searched by slightly moving the coil over the right M1 area until MEPs of maximal amplitude and lowest threshold in the left FDI were elicited. The exact coil position was marked by an inking pen. The stimulus intensity needed to evoke MEPs of approximately 0.8−1 mV peak-to-peak amplitude was defined (S1 mV). This intensity was used to evaluate MEPs changes before and after training and PAS protocols (see below). Twenty MEPs were recorded at each testing time. The peak-to-peak MEP amplitude on single trials was used to calculate the mean MEP amplitude On Day 0, MEPS were collected before PAS25 (PRE) after PAS25 (POST0) and 30 minutes after the application of PAS25 protocol (POST30). On Day 1 MEPS were collected before training (PRE training), after training (POST training) and after the application of PAS25 protocol (POST PAS25).

### Paired associative Stimulation

The PAS25 protocol consisted of electrical stimuli of the left median nerve at the wrist level paired with single TMS pulses over the hotspot of the FDI muscle area of the right hemisphere, delivered 25 ms later the electrical ones. Ninety paired stimulations were applied at 0.05 Hz over 30 min, with an inter-stimulus interval (ISI) of 25 ms. The TMS was delivered in the way described above, at S1 mV stimulus intensity. The electrical stimulation was applied through a bipolar electrode (cathode proximal) using a square-wave pulse (duration, 200 microseconds) at an intensity of three times the perceptual threshold (Digitimer D180 high voltage electric stimulator). Participants were instructed to look in front of them at a black screen, so as to standardize the visual attentional load during the PAS protocols^55^, and count the peripheral electrical stimuli they perceived. The MEPs evoked in the FDI were displayed online during the intervention to control for the correct coil position.

### Electromyographic (EMG) recording

EMG was recorded through surface electrodes from the left FDI muscle using pairs of Ag-AgCl electrodes. Electromyographic signals (EMG) were digitalized, amplified and filtered (20 Hz to 1 kHz) with a 1902 isolated pre-amplifier controlled by the Power 1401 acquisition interface (Cambridge Electronic Design Limited, Cambridge, UK), and stored on a personal computer for display and later offline data analysis. Each recording epoch lasted 400 ms, of which 100 ms preceded the TMS. Participants were constantly reminded to always keep their hand relaxed during the whole experiment. EMG signal was monitored visually by the experimenter and trials with background EMG activity were excluded from analysis.

### Power analysis

The main aim of the present study is to assess whether a training combining motor imagery and electrical stimulation is as effective in inducing plasticity in M1 as physical practice. We already showed that motor imagery training by itself induces changes in M1 plasticity (assessed as MEPs amplitude after the application of PAS25 protocol), that were minor than those induced by physical practice^18^. Therefore, we based our power analysis on the differences in M1 plasticity induced by physical practice in our previous study using similar methodology^18^. In the previous study we adopted a within subject design with a sample size of n = 9 participants who executed both physical practice and motor imagery training. This sample size yielded on 2-sided tests statistically significant within-group differences between MEPs amplitude after PAS25 protocol performed at rest or after physical practice session at a *p* < 0.04 level, with corresponding power of about 92%. Here, the experimental design involved three different trainings (physical practice, motor imagery and motor imagery combined with electrical stimulation) and we also tested the retention of skill learning 24 hours later. Thus, to exclude learning effect due to repetition of training sessions, we adopted a between-subjects design. On the basis of all these assumptions, we anticipated that a sample size of 12 subjects per group would be adequate to show statistically significant differences within each group on neurophysiological parameters explored (MEPs amplitude after PAS25 protocol performed at rest on Day 0 vs MEPs amplitude after PAS25 protocol performed after a training session on Day 1) with a power of about 92%.

### Data and statistical analysis

Data collected with the sensor-engineered glove were processed with a customized software. Finger opposition movements were described by: (i) movement rate, i.e. the number of contacts per second (Hz); (ii) touch duration (TD), i.e. the contact time between the thumb and index; (iii) inter-tapping interval (ITI), i.e. the time elapsing from end of contact between the thumb and index to the beginning of the subsequent contact. We considered the mean value between the two performances executed in the assessment phase before the training (Pre), immediately after the training (Post1) and the following day (Post2).

For movement rate, we also normalized data respect to baseline assessment (Pre) as follows:

Post1 = (movement rate Post1 − movement rate Pre)/movement rate Pre × 100)

Post2 = (movement rate Post2 − movement rate Pre)/movement rate Pre × 100)

Further, to measure also individual accuracy in increasing movement rate after training respect to the acoustic cue provided during training, we calculated, for each subject, the maximal rate provided by the acoustic cue and the performance rate after the training session and 24 hours later. Then, from these parameters we calculated the duration of the time interval set by the metronome (in ms, duration of the time interval between two successive acoustic cues, 1/Hz*1000) and the duration of the time interval reproduced (in ms, as processed by the customized GAS software as the sum of TD + ITI). From these parameters we calculated a “temporal accuracy index” as the difference between the reproduced interval minus the set interval, to test if the rate at post-tests was close to the rate of the acoustic cue.

We checked that all variables were normally distributed (Shapiro-Wilk W test) and that sphericity was respected (Mauchly tests). Raw motor performance data (Movement rate, ITI and TD) were separately entered in a RM-ANOVA with time (Pre, Post1 and Post2) as within subjects factor and with group (ESMI, MI and PP) as between subjects factor. Further, normalized movement rate and temporal accuracy index data were entered in a RM-ANOVA with time (Post1 and Post2) as within subjects factor and with group (ESMI, MI and PP) as between subjects factor.

To evaluate the effect of PAS25 on cortical excitability on Day 0 (baseline session), mean MEPs amplitude was subjected to a RM ANOVA with time (PRE, POST0 and POST30) as within subject factor and GROUP (ESMI, MI and PP). To evaluate the effect of the different trainings on M1 excitability and plasticity on Day 1, MEPs data were subjected to a RM ANOVA with time (PRE training, POST training and POST PAS25) as within subject factor and GROUP (ESMI, MI and PP) as between subjects factor. Further a Pearson correlation was performed between MEPs amplitude increase after PAS 25 on Day 0 (calculated as MEPs POST0- MEPs PRE/MEPs PRE*100) and MEPs amplitude increase on Day 1, after training session (calculated as MEPs POST training- MEPs PRE training/MEPs PRE training *100)

Statistical analysis was performed with SPSS 22.0. P-values of 0.05 were considered as threshold for statistical significance. *Post-hoc* analysis of significant interactions was performed by means of *t*-tests.
